# Yi Ai Fang, a traditional Chinese herbal formula, impacts the vasculogenic mimicry formation of human colorectal cancer through HIF-1α and epithelial mesenchymal transition

**DOI:** 10.1186/s12906-016-1419-z

**Published:** 2016-11-02

**Authors:** Fenggang Hou, Wen Li, Qi Shi, Hongjia Li, Shanshan Liu, Shaoqi Zong, Jianlin Ren, Jie Chai, Jian Xu

**Affiliations:** 1 Oncology Department of Shanghai Municipal Hospital of Traditional Chinese Medicine affiliated to Shanghai TCM University, Shanghai, 200071 China; 2 Department of Gastrointestinal Surgery, Shandong University Affiliated Shandong Cancer Hospital and Institute, 117 Jiyan Road, Jinan, Shandong 250000 China; 3 Mental diseases of Shanghai Municipal Hospital of Traditional Chinese Medicine affiliated to Shanghai TCM University, 274 Zhijiang Road, Shanghai, 200071 China

**Keywords:** Yi Ai Fang, HIF-1α, EMT, Colorectal cancer, Vasculogenic mimicry

## Abstract

**Background:**

Yi Ai Fang (YAF), a traditional Chinese medicine (TCM) formula, has been identified to have anticancer activity in our previously studies. The present study aimed to explore the potential mechanism of YAF suppression of VM on colorectal cancer (CRC) in vitro and in vivo.

**Methods:**

Cell viability was measured by CCK-8 assay. HIF-1α, E-cd(E-cadherin), Claudin-4, and VIM (Vimentin) expressions level in vitro were evaluated by Western blot or RT-PCR. In addition, Human CRC HCT-116 cells were implanted in BALB/c nude mice; mice with xenografted tumors were randomly administrated vehicle (control), 8, 16, or 32 mg/mL YAF, or 1 mg/mL fluorouracil (5-FU). HIF-1α, E-cd, Claudin-4, and VIM expression in these tumors were determined by IHC.

**Results:**

YAF effectively inhibited the growth and the formation of vasculogenic mimicry (VM) of CRC cells in a dose-dependent trend. YAF restrained the formation of vasculogenic mimicry(VM) through HIF-1α/EMT pathway in CRC. YAF suppressed VM was triggered by activation of E-cd and Claudin-4,which are characteristics of endothelial cells,and inhibition of HIF-1α and VIM in vitro. In vivo data showed that YAF remarkably inhibited growth of the xenografted tumors. The YAF-treated tumor samples were analyzed by IHC for levels of HIF-1α/EMT related proteins HIF-1α, E-cd, Claudin-4, and VIM. The results indicated that YAF significantly enhanced expression of E-cd and Claudin-4,but decreased expression of HIF-1α, VIM in a dose-dependent manner.

**Conclusions:**

In conclusion, this study provided the first direct evidence that YAF inhibited the formation of VM in human CRC, suggesting that YAF may be considered as a useful target for cancer therapy.

## Background

Colorectal cancer (CRC) is the third most common cancer worldwide [1]. More than half a million people die from colorectal cancer each year. The incurable factor of CRC is tumor metastasis, in which blood supply was necessary. In other words, without angiogenesis, tumors cannot grow beyond 2–3 mm [2]. Therefore, the antiangiogenic drug bevacizumab (Avastin) has become one of the most popular vascular-targeted therapeutic drugs for several types of tumors, including CRC [3]. However, this traditional antiangiogenic drug can accelerate metastasis at the same time, together with marked hypoxia and an alternative blood supply vasculogenic mimicry (VM). Tumor cells mimic endothelial cells VM to consists of laminin-rich networks that can be stained with periodic acid-Schiff (PAS) in vivo and forms extracellular matrix (ECM)-rich tubular networks on Matrigel that mimic conventional angiogenesis in vitro [4]. That is to say,the formation of VM lead to the failure of vascular-targeted therapy and the metastasis of CRC.

Epithelial-mesenchymal transition (EMT) is a dynamic biological process where polarized epithelial cells close their epithelial characteristics and show phenotypes of mesenchymal cells. Growing evidence suggests that, epithelial tumor cells capable of VM imitate endothelial functions and display some endothelial properties typical of mesenchymal cells [5, 6]. Therefore, we hypothesized that VM formation in epithelial cancer is associated with the EMT process, and the regulators that contribute to EMT may also modulate VM formation.

The hypoxic microenvironment of tumors regulates the proliferation, angiogenesis, and metabolism of cells modulated by hypoxia-inducible factor-1α (HIF-1α) in vivo and forms extracellular matrix (ECM). Hypoxia provides tumor cells with greater resistance to anticancer therapies through EMT [7]. Further more, HIF-1α affects the expression of EMT marks, such as E-cd, Claudin-4, and VIM [8]. If HIF-1α is a key inducer that contributes to tumor EMT, the function of HIF-1α in VM formation in CRC should be investigated. Both hypoxia and HIF-1α activation in tumor angiogenesis are illuminated, but their roles in regulating VM are still puzzling.

Yiai Fang is a compound Chinese medicine guide by TCM principles, which is composed of Astragalus membranaceus (Huangqi), Atractylis ovate (Baizhu), Actinidia arguta (Tengligen), Curcuma zedoaria (Ezhu), and Benincasa hispida (Dongguazi). The major pharmacologic components are astragaloside IV (ASIV, from Huangqi), Atractyloside III(ATRIII, from Baizhu), Isocurcumenol (from Ezhu), Oleanolic Acid (from Tengligen), Trigonelline (from Dongguazi). Chinese herbal medicine have been used to fight cancer for a long time [9, 10]. Astragalus membranaceus have been used to treat ovarian cancer, showed clinical efficacy in treating ovarian cancer clinical trial [11]. Atractylis ovate play an important role in control of pancreatic cancer [12]. Antitumor effects of isocurcumenol isolated from curcuma zedoaria on human and murine cancer cells were proved in study [13]. Oleanolic acid extracted from actinidia arguta,inhibit tumor gene expression in cancer cell [14]. In addition, our previous studies showed that Yiai Fang is effectively to control CRC progression, improve quality of patients life, and prolong survival times [15].

Yiai Fang had been found to have an anti-cancer effect, however, there are few well-controlled scientific studies on the mechanisms of action of this prescription used to treat CRC. In this study, our aim was to investigate the effect and the molecular mechanism of Yiai Fang in CRC both in vitro and in vivo.

## Methods

We confirmed that the Ethical Committee of the Shanghai University of Traditional Chinese Medicine approved all the experiments described in this paper.

### Preparation of drugs

Drugs were purchased from Jiangyin tianjiang pharmaceutcal co., LTD (Jiangsu, China). Herb was boiled in water according to the traditional method of herbal tea preparation. The filtrates were concentrated and dried in vacuum at 60 °C. The concentrated extract was then dried by lyophilization to obtain the YAF extract at a yield of dried powder of 26.5 %. The content of its major components was detected by high performance liquid chromatography (HPLC), purity: 99 %. The proportions of Astragalus membranaceus (Huangqi), Atractylis ovate (Baizhu), Actinidia arguta (Tengligen), Curcuma zedoaria (Ezhu), and Benincasa hispida (Dongguazi) in YAF are 40, 20, 5, 2,6 %, respectively. YAF were dissolved in PBS (Gibco Life Technologies, USA) to make a solution. The extract was stored at 4 °C, and it was standardized, regulated, and qualified according to the guidelines defined by Chinese State Food and Drug Administration (SFDA).

### Experimental animals

All animal studies were approved by the ethics committee of the Putuo Hospital affiliated to Shanghai University of Traditional Chinese Medicine and the principles of laboratory animal care were followed in all animal experiments. A total of 48 BABL/c nude male, 18 ± 2 g, SPF grade, were purchased from Shanghai Slac Laboratory Animal Co.Ltd (Shanghai, China, number of animal license SCXK (Shanghai) 2008-0016), and were fed in the experimental animal room in Putuo Hospital affiliated to Shanghai University of Traditional Chinese Medicine (Shanghai, China, number of animal laboratory license, SCHK (Shanghai) 2007-0005). Sixteen animals were randomly allocated into 2 groups prior to the experiment. Human colorectal cancer HCT116 cells were used to establish the xenografts, which were resuspended at a density of 1 × 10^7^/ml. The suspension (0.1 ml/10 g body weight) was injected subcutaneously into the nude mice. After 12 days, mice with unbroken transplanted tumor were sacrificed, and using the above-described method, the tumors were re-implanted and when they reached a size of 50–100 mm^3^, the tumor-bearing mice were randomly divided into five groups (six mice for each group): the control group (isometric normal saline), the YAF groups (8, 16, and 32 mg/kg/day, respectively) and the fluorouracil (5-FU) group (1 mg/kg/day). All the drugs were given by gavage administration. The Length and the width of the tumor were measured and the volume was calculated using the formula: Volume = 1/2(width^2^) × length. On day 21, the mice were sacrificed and the tumors formed were extracted and weighed after carefully removing the extraneous tissues. They were fixed in buffered formalin and processed for paraffin sectioning.

### Cell lines and culture

Human CRC cell line HCT-116 (isolated from a primary colonic tumor) was purchased from the Shanghai Cell Collection (Shanghai, China). HCT-116 cells were cultured in Roswell Park Memorial Institute (RPMI) 1640 medium supplemented with 10 % fetal bovine serum (FBS), 1 mM glutamine, 1 % penicillin/streptomycin, HT-29 was cultured in McCoy’s 5A medium with 10 % FBS (all of these were purchased from Gibco Life Technologies). All cells were cultured with 100 μg/mL streptomycin (Invitrogen, Carlsbad, CA, USA) at 37 °C in a 5 % CO_2_ humidified incubator (Thermo Fisher Scientific Inc., Waltham, MA, USA).

### CD31/PAS double staining

CD31 immunohistochemical staining was applied on the sections before PAS staining. The slides were rinsed with distilled water after diaminobenzidine was used as the chromogen, then treated with 0.5 % periodic acid solution for 10 min, and rinsed with distilled water for 2–3 min. In a dark chamber, the slides were treated with Schiff solution for 15–30mins. After distilled water rinsing, sections were counterstained with hematoxylin.

### Cell viability by CCK-8 assays

The cell viability was detected using the CCK-8 assay. That is to say, cells were seeded into 96-well plates at a density of 1 × 10^5^ cells/well and stabilized for 24 h at 37 °C before being treated with Yiai Fang 0, 25, 50, 100, 150 and 200 μg/ml of for 48 h. The concentration of 0.1 % dimethyl sulfoxide (DMSO,solvent) was used for the control regimen. Then,100 μl of 0.5 mg/ml CCK-8 solution was added to each well. After 2 h,the formazan precipitate was dissolved in 100 μl DMSO, and then the absorbance OD values were measured in a multiscan plate reader (Varioskan Flash, Thermo, CA, USA) at 456 nm. Cell viability of the control cells treated with 0.5 % DMSO was set as 100 %. The percentage viability was calculated as: % Viability = [OD of treated cells/OD of control cells] × 100. The CCK-8 test was repeated in triplicate.

### Three-dimensional cultures

VM formation was tested by using 3-D culture containing rat tail collagen type I in vitro. The 6-well plates were pretreated by a certain percentage of the rat tail collagen type I for 30 min. Then 4 × 10^5^ tumor cells were plated onto the surface of collagen and incubated at 37 °C. For in-gel methods, the tumor cells were mixture-seeded with Matrigel to allow them to polymerize. Filtered CoCl_2_ was added to the medium in the hypoxic group 48 h after polymerization.

### Real-time quantitative PCR

Total cellular RNA was isolated using Trizol reagent (Invitrogen Life Technologies) according to the manufacturer’s protocol. RT reactions were done using the Takara Kit under the instruction of the protocol. Quantitative PCR for target RNAs was performed using the SYBR green system (Takara). The forward primer for HIF-1α was 5’-CATCTCCATCTCCTACCCACAT-3’, and the reverse primer was 5’-ACTCCTTTTCCTGCTCTGTTTG-3’. The forward primer for human Vimentin was 5’-GAA GAGAACTTTGCCGTTGAAG-3’, and the reverse primer was 5’-ACGAAGGTGACGAGCCATT-3’.

The forward primer for human E-cadherin was 5’-GTCTCTCTCACCACCTCCACAG-3’ and the reverse primer was CAGACAGAGTGGGGAAAATGTA-3’. The forward primer for Claudin-4 was 5’-AAGTGACAGGGTGTGGTGGT-3’ and the reverse primer was5’-CAGACAGAGTGGGGAAAA TGTA-3’. Data are expressed as gene expression level relative to β-Actin (forward primer, 5’-AGCGAGCATCCCCCAAAGTT-3’; reverse primer, 5’-GGGCACGAAGGCTCATCATT-3’). All experiments were performed in triplicate and had a non-template reaction as a negative control. Relative mRNA expression levels were calculated using the 2-ΔΔCt method.

### Western blotting analysis

Before extracting protein, HCT-116 cells were treated with Yiai Fang for 24 h. The protein was extracted from the cultured cells and then homogenized with a protein extraction kit (Applygen Technologies, Beijing, China) on ice, according to manufacturer’s instruction. The whole protein was then separated on 12 % SDS-PAGE and transferred to NC membrane. The membrane was incubated overnight at 4 °C with antibodies, respectively, against HIF-1α (1:1000, Novus Biologicals, USA), β-actin (1:5000, Cell Signaling Technology, USA), E-cd (1:5000, Cell Signaling Technology, USA), Claudin-4 (1:2000, Cell Signaling Technology, USA), and VIM (1:2000, Cell Signaling Technology, USA). Then,the membranes were incubated with secondary antibody for 24 h at 4 °C, and immunoreactive bands were revealed using an enhanced chemiluminescence system. The protein signal in the X-film was quantified by scanning densitometry and further evaluated by a bio-image analysis system (Quantity One image analyzer software, Bio-Rad, Richmond, CA, USA).

### Immunohistochemistry (IHC)

The tumor tissues were fixed in 4 % paraformaldehyde for 48 h. Then they were embedded in paraffin and made into 4 μm slides. Tissue sections were processed by de-paraffining, rehydrating through an alcohol gradient, peroxidase clearing, antigen retrieval and blocking, antibody binding, DAB staining, washing with distilled water, hematoxylin staining, niacin alcohol differentiation, dilute ammonia bluing, incremental graded alcohol dehydration, xylene and conventional resin mounting. The primary antibody was rabbit-anti-human HIF-1α monoclonal antibody diluted by 1:50 (Abcam, Cambridge, MA, USA) rabbit-anti-human monoclonal antibody E-cadherin (Cell Signaling, USA, dilution 1:100) Claudin-4 (Santa Cruz, USA, dilution 1:100) and Vim (Affinity, USA; dilution 1:100) overnight at 4 °C. The secondary biotin-labeled antibody was used at 1:200. Positive blue staining for CD31 was visualized by Light yellow or tan substrate and cell nuclei (blue) were counterstained by hematoxylin. Under 400 × magnification, five random fields were selected. Staining was assessed as: non-staining: 0 point; light brown: 1 point; brownish yellow: 2 points; and dark brown: 3 points. Percentages of positive-stained cells were rated as: positive cells ≤5 %: 0 point; 6–25 %: 1 point; 26–50 %: 2 points; and ≥75 %: 3 points. Points for staining and percentage were multiplied for a 10-point scale: 0 point: negative (−), 1–3 points: weakly positive (+); 4–6 points: positive (++); and 7–9 points: strongly positive (+++) [16].

### Statistical analysis

All the results were expressed as the mean ± SD. If data met the homogeneity of variance of Gaussian distribution, one-way analysis of variance will be used for statistical inference; otherwise, non-parametric tests will be used. A *P* value of <0.05 was considered statistically significant.

## Results

### YAF inhibited proliferation of HCT-116 cells and depressed the information of VM in HCT-116 cells

**Fig. 1:**
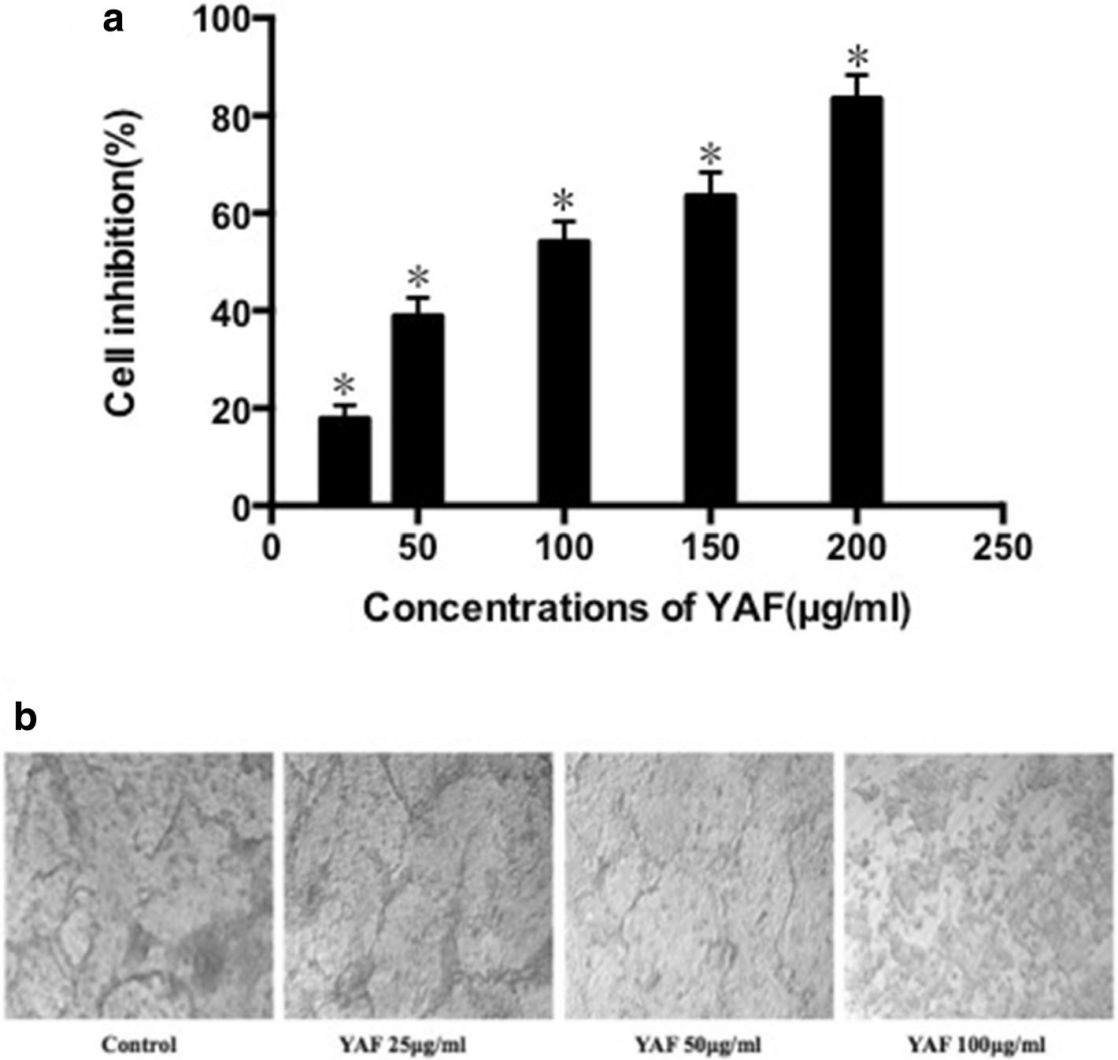
**a**. YAF reduces the viability of HCT-116 cells. The cells were incubated with YAF at 0, 25, 50, 100, 150 and 200 μg/ ml for 48 h. The viable cells were determined using CCK-8 assay. Data presented were means ± SD of three independent experiments. *P < 0.05 compared with the control group. **b**. VM capacity in vitro in colorectal cancer cells is affected by YAF. HCT116 cells were treated with YAF at 25, 50 and 100 μg/ml for 48 h, the constitution of VM was significantly decreased by YAF compared with that of untreated cells (*P < 0.05)

The antitumor effects of YAF on HCT-116 cells were evaluated by CCK-8 assay. As is shown in Fig. 1a, YAF inhibited proliferation of HCT-116 cells in a dose-dependent manner. After 48 h of treatment,the average IC50 of YAF against HCT-116 cells was 79.27 μg/ml. VM is a type of angiogenesis that is accompanied by a specialized series of cellular events such as matrix reconstruction and cell migration [17]. To quantify the VM in HCT-116 cells, cells were stained with CD31/PAS, and then subsequently analyzed by microphotograph. What’s more, the formations of VM in HCT-116 cells were affected by YAF in a dose-dependent fashion too. After treatment with YAF at 25, 50 and 100 μg/ml for 48 h, the constitution of VM was significantly decreased by YAF compared with that of untreated cells (*P* < 0.05) (Fig. 1b). Taken together, YAF inhibits the formations of VM in HCT-116 cells at concentrations much lower than those required to inhibit HCT-116 cells.

### YAF impact VM formation through HIF-1α and EMT in HCT-116 cells

**Fig. 2:**
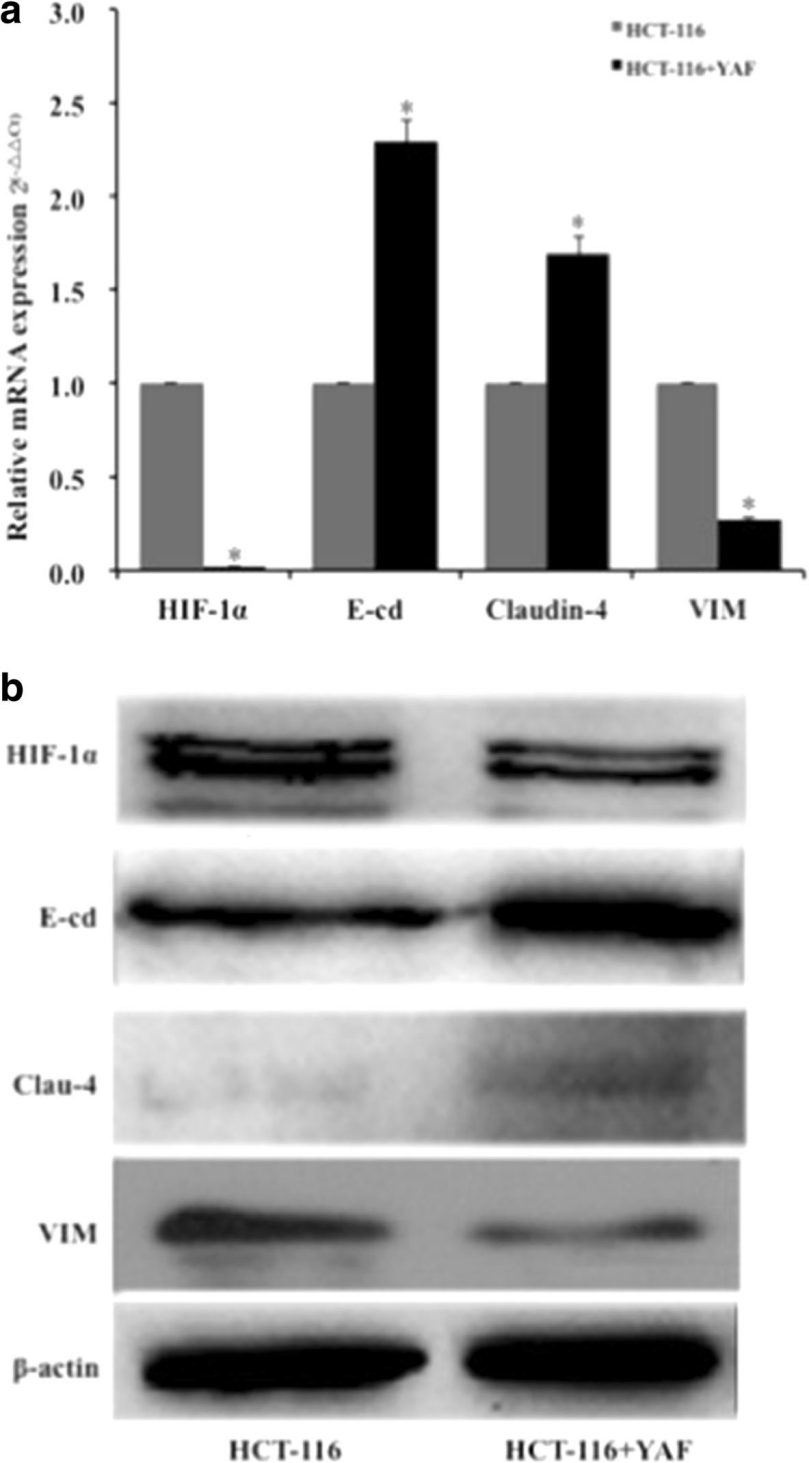
YAF influences HIF-1α and EMT expression analysis in HCT-116 cell lines. **a**. Cells were incubated for 48 h in YAF. The level of HIF-1α, Clau-4, E-cd and VIM mRNA were found. **b** HCT-116 cells were incubated with YAF for 48 h. The level of HIF-1α, Clau-4, E-cd and VIM protiens were found. *P < 0.05. Results are folden change ± SE of at least three independent experiments

In the process of VM formation by tumor cells, HIF-1α and EMT play an important role. To further verify this point and research the effect of YAF, HCT-116 cells were treated with YAF. Closer investigation the results of Western blotting and qPCR in CRC cells showed that YAF inhibited HIF-1α and VIM expression, but increased E-cd and Claudin-4 levels (Fig. 2) compared with the control group. The data come from CRC cells, which were incubated with YAF at 25 μg/ml for 48 h. This suggests that YAF hinders the formation of VM in CRC cells through HIF-1α and EMT dependent mechanism.

### YAF prevents VM formation by influence HIF-1α and EMT in CRC xenograft tumors

**Fig. 3:**
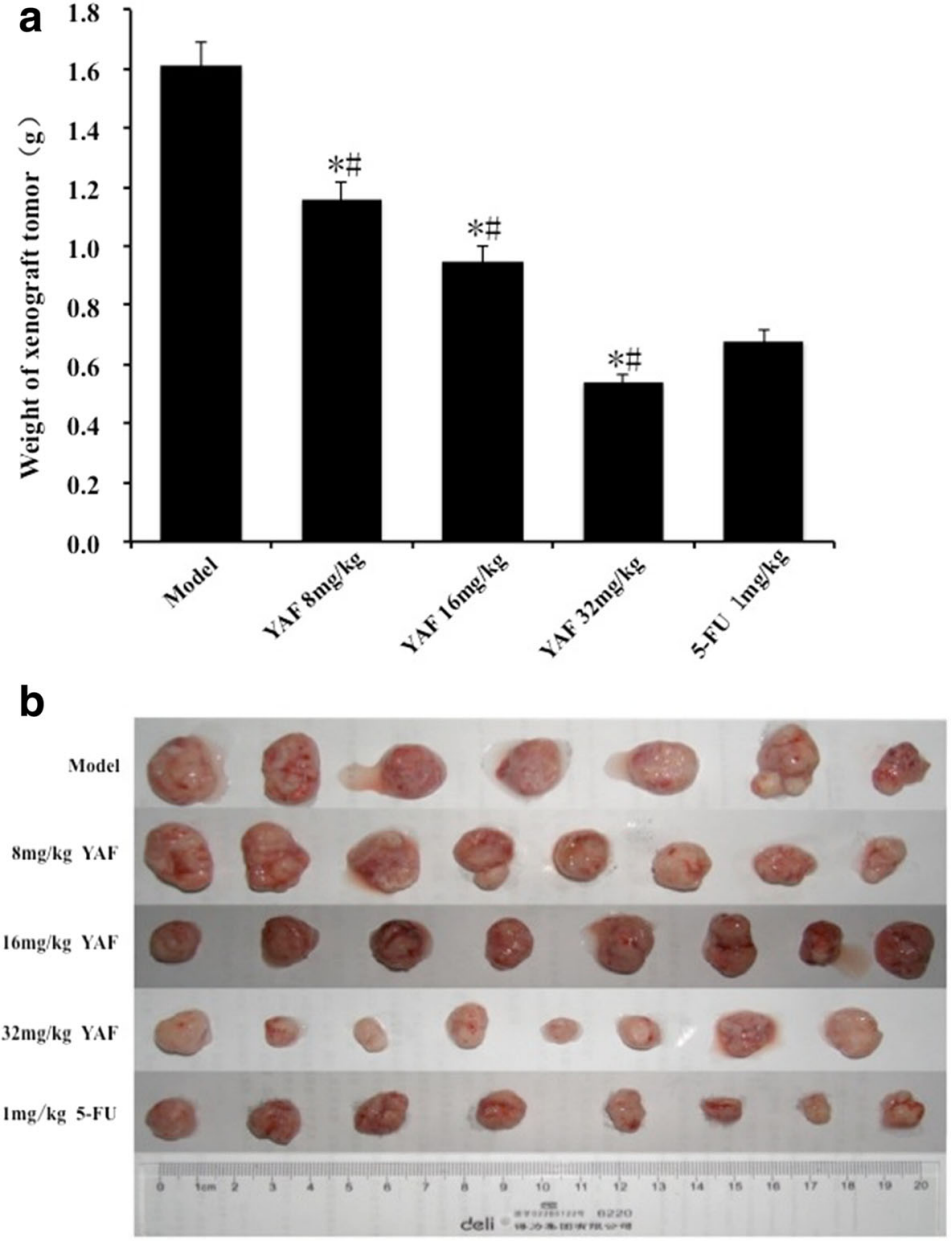
Inhibition effect of YAF in vivo. **a** Tumor weight change was determined every two days during delivery period. Tumor weight inhibition rates in the low-, medium-, and high-YAF dose groups were 27.95, 40.99, and 66.46 %, respectively. Values are mean weight of nude mice ± SD. Statistical difference was analyzed by Student’s *t*-test. *P < 0.05 compared with that of control group. **b** Tumors removed from nude mice and photographed on the 21th day after administration

**Fig. 4:**
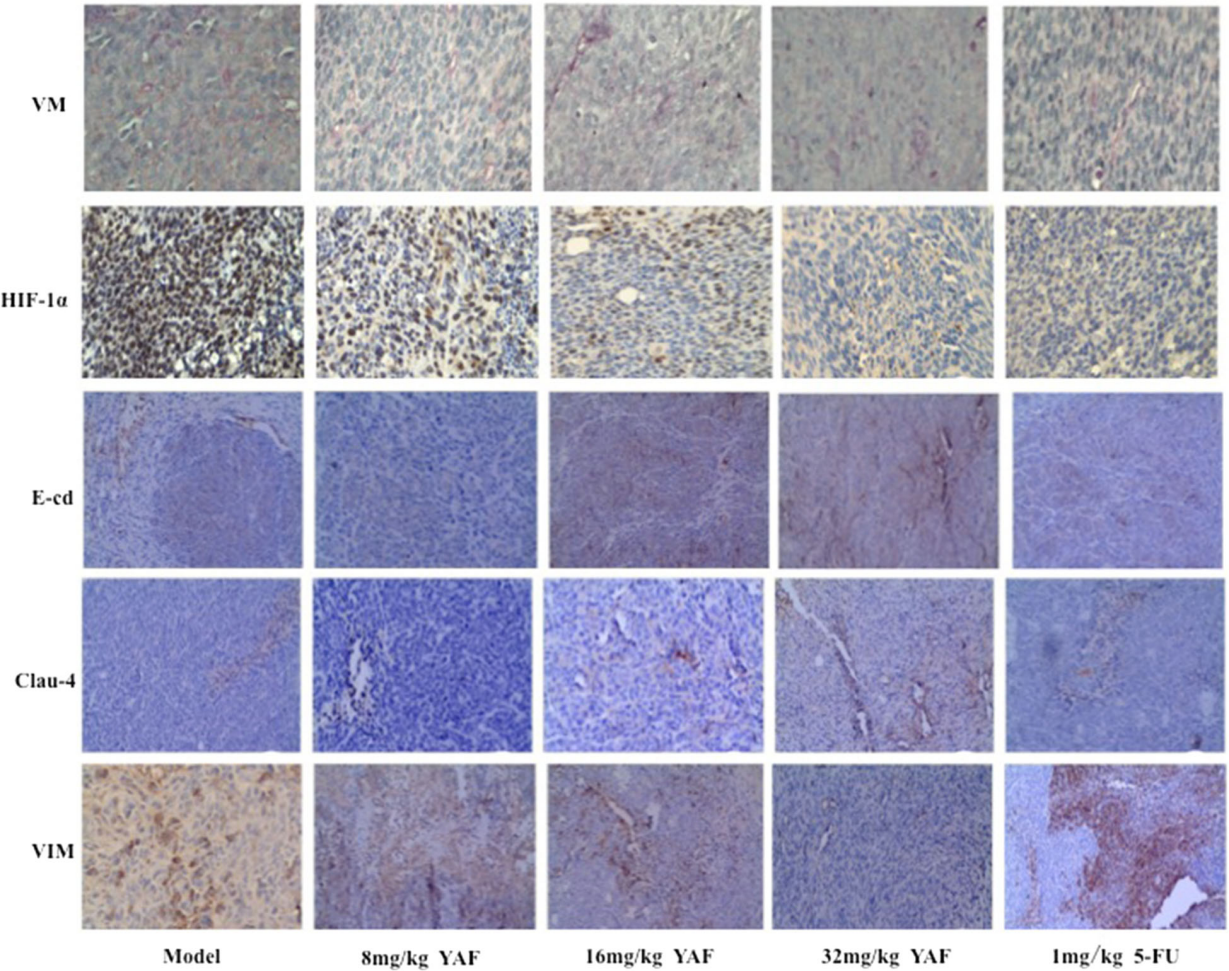
VM formation is association with HIF-1α and EMT markers in CRC xenograft tumors. The IHC of CRC xenografts vivo data showed that HCT-116 VM formation was inhibit by YAF. The effect of YAF on HIF-1α and E-cadherin, Claudin-4 as well as Vimentin, in CRC xenograft tumors

In order to confirm the effects of YAF for CRC in vivo, we established the human CRC xenograft mode in nude mice, which were then treated with different doses of YAF(8,16 and 32 mg/kg/day for 14 day). The positive control group were treated with 5-FU(1 mg/kg/day for 14 day). Our data showed that YAF remarkably inhibited growth of the xenografted tumors (Fig. 3a and b). Unfortunately, one mouse in control group died for unclear reason dury our research, but this doesn’t disturb our results. Tumor weight inhibition rates in the low-, medium-, and high-YAF dose groups were 27.95, 40.99, and 66.46 %, respectively. Obviously, at the highest doses, the anti-tumor effect of YAF was similar to that of 5-FU (57.74 %). The xenograft tumor samples were then analyzed by IHC for levels of proteins such as HIF-1α, E-cd, Claudin-4, and VIM. The results demonstrated that YAF significantly enhanced expression of E-cd and Claudin-4 proteins in tumors, but decreased expression of HIF-1α and VIM in a dose-dependent manner (Fig. 4).

## Discussion

In 1999, vascular mimicry was reported in melanoma. The found of VM explained that tumor cells have more than one “trick” to ensure that they receive nourishment [18]. This finding demonstrated why a lot of drugs once heralded as a panacea in cancer treatment was less effective than hoped [19]. Cancer cells have infinite plasticity, which enable them to alter their cell markers, and adapt to specific microenvironments [20]. Hypoxia (or induction of HIF-1α), which plays an important role in microenvironment in induces EMT, and promotes the formation of VM [21]. Exploring the mechanisms of VM will increase our understanding of cancer development, and may identify new therapeutic approaches. Therefore, HIF-1α and EMT were the key factor, that control VM.

Several preliminary work showed that VM exists in CRC, and VM formation have relationship with HIF-1α and EMT. It was well konwn that,traditional Chinese medicinal herbs have been used to treat cancer for a lot of years. In recent research, it has been found that VM in cancer can be inhibited by the medicine. However, the specific function of YAF was unclear in CRC VM prior to the present study. Our in vitro data showed that YAF effectively suppresses metastatic CRC cell HCT-116 dominant vasculogenic mimicry, which is associated with the inhibition of HIF-1α and VIM genes expression and cell exposure of the proteins. In contrast, E-cd and Claudin-4 levels both in mRNA and protein were increased by YAF. Xenograft HCT-116 tumors in BALB/C nude mice were applied for further investigating the mechanisms underlying the inhibiting effect of YAF on CRC. The present data demonstrated that YAF can significantly shorter tumor volume and decrease tumor weight. At this time, HIF-1α and VIM appeared to be affect by YAF at the pattern same with in vitro. What’s more, Furthermore, CRC cells instructed VM to produce in E-cd and Claudin-4 independent manner.

## Conclusions

In conclusion, this study firstly observed the correlation among observed the correlation between HIF-1α, EMT and VM in CRC intervened by though YAF. Our data strongly imply that YAF inhibited VM both in vitro and in vivo. The HIF-1α and EMT have an important role in the development of VM, and in the case of cancer. The traditional Chinese medicine YAF, has been found to have implications for the rational development of novel regimens in human CRC.
